# Appraising the Factors Associated with Delirium Care Behaviours and Barriers to Their Assessment Among Clinical Nurses: A Cross-Sectional Study

**DOI:** 10.3390/ijerph21121582

**Published:** 2024-11-27

**Authors:** Susan Ka Yee Chow, Soi Chu Chan

**Affiliations:** 1 Discipline of Nursing & Healthcare, Hong Kong Nang Yan College of Higher Education, Hong Kong SAR, China; 2 Faculty of Medicine, Macau University of Science and Technology, Macao SAR, China; 3 Kiang Wu Hospital, Macao SAR, China; tiffanychan192837qaz@hotmail.com

**Keywords:** delirium, nurse, knowledge, behaviour, barriers

## Abstract

Delirium can occur at any age, although the incidence is higher in older patients and after surgery. Although delirium is an acute, potentially reversible, cognitive disorder, there is evidence that it is associated with increased healthcare costs and imposes a significant burden on patients, families, hospitals, and public resources. The aim of this study was to investigate and assess the knowledge, behaviours, and factors influencing assessments of delirium by hospital nurses so as to predict the factors associated with their current delirium management behaviours. A cross-sectional survey was conducted among 342 nurses in different hospitals in Macau. The questionnaires included items on the respondents’ demographic information, knowledge of delirium care, nursing behaviours, and factors influencing nurses’ assessment of delirium patients in their daily practice. The descriptive statistics showed that nurses were found to have a moderate level of knowledge about the management of delirium. The repeated measures ANOVA revealed that patient factors were the most significant, outweighing individual and organizational factors as barriers to assessing patients with delirium. The Pearson’s correlation showed a moderate positive correlation between delirium care knowledge and delirium care behaviour (r = 0.339). With regard to factors influencing delirium care behaviours, multiple linear regression models showed that the significant predictors were years of work experience (β = 0.206, 95% CI: 1.125–3.158), the duration of delirium care courses (β = 0.103, 95% CI: 0.118–3.339), the knowledge of delirium care (β = 0.264, 95% CI: 0.474–1.019), and personal factors influencing nurses’ delirium assessments (β = −0.239, 95% CI: −1.031–−0.432). To enhance delirium management and achieve the optimal care of patients with delirium, formal education and training are crucial. Organizations should develop structured protocols and workflows that empower nurses. By integrating organizational strategies with individual efforts, clinical practices can be improved, resulting in optimal delirium care for patients.

## 1. Introduction

Delirium is an acute, reversible, and cognitive disorder in which patients experience varying degrees of altered consciousness and mental status [1]. Risk factors for the development of delirium in patients are associated with advanced age (age ≥ 65 years). In internal medicine and geriatric wards, the incidence of delirium in those aged ≥ 65 years has been found to be about 29–64% [2]. It is estimated that the proportion of elderly people who undergo anaesthesia and surgery will exceed 50% by 2051 [3]. About 10–15% of elderly patients seeking emergency care develop delirium [4], and the incidence of delirium in terminally ill cancer patients can be as high as 85% [5].

Macau is a special administrative region of the People’s Republic of China. The latest census data, taken from the end of 2023, indicate that the total population of Macau is 683,700, and that 14.0% of the population is above the age of 65 [6]. In accordance with the World Health Organization, a country is classified as an ‘aged society’ when the proportion of individuals aged 65 or above reaches a level of between 7% and 14% [7]. With advanced technology and enhancements in medical care, the average life expectancy in Macau is projected to reach 84 years of age, leading to a significant prevalence of delirium among the population.

The clinical presentation of delirium varies between different types of delirium. Hyperactive delirium is the most recognizable type of delirium, as those with this condition become agitated and hypervigilant. Patients with hypoactive delirium present with lethargy or decreased activity. The signs may fluctuate between these two types of delirium as the symptoms are not easily recognized [8]. The onset of delirium can lead to a number of adverse outcomes, including death and debilitation. Without the appropriate management of symptoms or interventions, patients may injure themselves and others, experience unplanned extubation, perform the self-removal of catheters, experience unintentional falls, develop pressure sores, display a decrease in physical functioning, and require a prolongation of hospitalization [9]. The consequences of this are increased hospitalization costs, which place a tremendous burden on patients and families, hospitals, and public resources. Surveys have shown that the estimated medical costs per delirium patient range from USD 16,303 to USD 64,421, leading to USD 152 billion in healthcare system expenditures [10].

The prevention of delirium in clinical practice involves early detection and effective management at an early stage. Delirium care management should include the identification of possible causes, the correction of the etiology of delirium, and the management of delirium symptoms through pharmacological and nonpharmacological treatments [11]. Given the close relationship between nurses and their patients, which encompasses both the provision of care and the evaluation of health outcomes, it is of the utmost importance that nurses receive training in the early assessment, risk identification, and appropriate care of patients with delirium to ensure the maintenance of patient safety and quality of care [12,13].

### Knowledge and Caring Behaviour of Nurses When Caring for Patients Experiencing Delirium

With regard to nurses’ knowledge of delirium care, a survey of 130 nurses across various hospital wards in New Zealand revealed a high level of awareness, with 80% of respondents demonstrating a good understanding of the subject matter. Significant differences in scores were observed between nurses of different job ranks [14]. In contrast, a recent study in Cyprus, which examined both the knowledge and attitudes of 558 nurses in acute care hospitals, found that nurses demonstrated limited knowledge of acute delirium. However, there was a correlation between the nurses’ knowledge and their attitude [15]. In South Korea, the self-reported knowledge of delirium care among intensive care unit (ICU) nurses and ward nurses was moderate, with scores of 57.1% and 60.7%, respectively [16]. A multicentre cross-sectional study in China revealed that the knowledge and practice of nurses in delirium care require further improvement. The total scores for knowledge and attitude were affected by age, ward unit, job title, and familiarity with delirium [17]. In Spain, approximately half of the nurses demonstrated sufficient knowledge, while the primary barriers were largely institutional, including a lack of training and a lack of standardized protocols [18]. A recent qualitative systematic review and meta-aggregation of nurses’ experiences in managing the delirium of older patients in acute settings revealed that various stakeholders in an organization, extending from organizational leaders to colleagues, constituted the primary barriers influencing nurses’ behaviours in delirium care [19].

In terms of barriers to delirium assessments, a literature review found that the individual barriers were a lack of competence and knowledge, or unfamiliarity with the patient’s condition and the assessment tools. The patient-related barriers included difficulty in assessing sedated patients and higher nurse-to-patient ratios, but the most significant factor for ICU nurses in performing delirium assessments was the complexity of the condition of the patients in the unit. The complexity of the condition of patients and sedated patients in the intensive care unit was the determining factor in delirium assessment [20,21,22]. When comparing barriers between ICU and ward nurses in delirium care, ward nurses were found to experience more emotional distress and exhaustion due to patient agitation [17,23]. A qualitative study conducted in China revealed that the barriers to assessment by a geriatric specialist included language, the experience of postoperative assessments, and the misperceptions of delirium assessments held by elderly relatives of the patient [24]. ICU nurses in southwest China reported that the greatest barriers to assessing subsyndromal delirium were those prevailing at the patient level, including those related to intubated and sedated patients [23]. The literature review found that most studies on barriers to conducting delirium assessments focused mainly on ICU nurses or nurses in specialty units, and rarely on the whole population of nurses working in different clinical settings.

In general, there were wide variations in nurses’ knowledge and behaviours and in the three dimensions of factors influencing assessments of delirium in daily patient care. Most importantly, previous studies mostly focused on critical care nurses, with no studies conducted on the entire cohort of nurses with regard to their overall knowledge of delirium, details on their caring behaviour, and the barriers that they face to assessing patients in clinical settings. There is a paucity of research on barriers that influence the behaviour of nurses from individual, patient, and organizational perspectives. In terms of academic research in Macau, there have been few studies on the delirium care provided by nurses, despite the ageing population. In this study, we investigated nurses’ delirium knowledge and caring behaviours, as well as the barriers that they face when conducting delirium assessments for patients. In filling this knowledge gap, the broad perspective taken in this study could contribute to improving evidence-based nursing practice for hospitalized patients.

Based on the background information, our research questions are as follows:

1. What is the current state of nurses’ knowledge and practice of delirium care?
2. What is the relationship between nurses’ delirium care knowledge, caring behaviours, and barriers to conducting delirium assessments?
3. What are the factors that prevent nurses from performing delirium assessments?
4. Are nurses’ personal variables predictive of clinical delirium care behaviour?

## 2. Materials and Methods

A cross-sectional survey design was employed in this study, utilizing an electronic questionnaire distributed among the various nursing associations in Macau. The criteria for inclusion were nurses with a valid certificate and a full-time engagement in hospital-based practice. Excluded were nurses serving internships and those working in emergency rooms, outpatient departments, and dialysis units.

### 2.1. Sample Size Calculation

According to the Statistics and Census Bureau of the Macao Special Administrative Region, there were 1932 nurses working in the hospitals [25]. The sample size was calculated according to the work of Rea and Parker [26]. The following formula was used to calculate the size of the sample required for this study as a proportion of the total population:

$$S=\frac{z_{a}^{2}(0.25)(N)}{z_{a}^{2}(0.25)+\left(N-1\right){ME}_{p}^{2}}$$

$$S=\frac{1.96^{2}\left(0.25\right)\left(1932\right)}{1.96^{2}\left(0.25\right)+\left(1932-1\right)(0.05^{2})}$$

In statistical analysis, the required sample size (S), population size (N), confidence level (Z), and margin of error (M) are fundamental parameters. In this context, Z represents the confidence level, set at 95% (Z = 1.96), while M denotes the margin of error, fixed at 0.05. We used a sample size of 320 subjects in accordance with the random sampling formula. Considering the possibility that 5% of the questionnaires in the study would be substandard, the estimated sample size of the current study was set to 340 participants.

### 2.2. Research Questionnaire

The study’s instrument comprised a general information questionnaire on demographics, a delirium knowledge questionnaire, a questionnaire on nurse behaviour, and a questionnaire on factors influencing the assessment of delirium in nurses.

The researchers devised a questionnaire on demographics. The delirium knowledge questionnaire used in this study was developed by Wang [27]. The questionnaire comprises 13 items pertaining to the clinical symptoms of delirium and 17 items related to risk factors. A score of one was awarded for each correct answer, with incorrect responses and a lack of knowledge counted as zero points. The total score ranged from 0 to 30 points, with higher scores indicating better knowledge. The content validity was 0.94, and the split-half reliability was 0.75, indicating that the instrument exhibits good validity and internal consistency.

The Delirium Care Behaviour Questionnaire was developed by Wang [27]. The questionnaire was constructed based on three dimensions, and examined nurses’ behaviours in the context of delirium prevention, assessment, and care. The 20 questions were scored on a 5-point Likert Scale, ranging from ‘never’ to ‘often’. The scores were converted to a scale of 20 to 100, with higher scores indicating greater accuracy and appropriateness in terms of the implementation of the practices of the study participants. The content validity was 0.99, and the Cronbach’s alpha coefficient was 0.93, indicating good validity and internal consistency. 

The scale developed by Wu, Li, and Zhu [28] was employed to investigate the factors influencing the assessment of delirium in routine patient care. The scale comprises three dimensions: the individual perspective (7 items), the organizational perspective (8 items), and the patient perspective (6 items). The 21 items were scored on a 5-point Likert Scale, ranging from ‘never’ to ‘often’. The scores ranged from 21 to 105, with higher scores indicating greater hindrance. The content validity of the three dimensions ranged from 0.82 to 0.91, and the Cronbach’s alpha was 0.79, indicating good validity and reliability.

To ensure that the three questionnaires were culturally relevant to the nursing population in Macau, the authors requested the assistance of five experts, namely, academics, experienced medical practitioners, and nurses, to conduct a content validity test of the study’s scale. Following the input of these experts, a few items were revised to reflect their comments. Not only did the revisions improve the clarity of the questionnaires, but they also made the language in the survey more colloquial for Macau-based respondents. To ensure the stability of the scales, test–retest reliability was determined with 15 respondents who met the criteria for inclusion in the study. The intra-class correlation of the questionnaire on delirium knowledge was 0.762; that of the delirium behaviour questionnaire was 0.973; and that of the factors affecting the assessment of delirium was 0.968. These results indicate that the modified questionnaires had a high level of stability and reliability [29].

### 2.3. Data Collection

The data were collected in early 2023. Because of objective factors related to administrative procedures in the hospitals, the confidentiality of the hospitals’ information, and limited manpower resources, the sampling method used in this study was convenience sampling, performed via the social network groups of the nurses’ organizations. A total of 376 questionnaires were distributed, from which 342 completed questionnaires were received, representing a response rate of 90.95%.

### 2.4. Data Analysis

The SPSS Statistics software, version 28.0 (IBM Corp., Armonk, NY, USA), was used to analyze the data. Descriptive analyses were conducted to describe the basic demographic characteristics of the participants, their delirium knowledge, the delirium care behaviour, and the factors influencing their assessment of delirium. A Pearson’s correlation test was conducted to test the correlations between the aforementioned variables. A repeated measures ANOVA was applied to determine the differences in the scores of the three dimensions of factors influencing the assessment of delirium in daily patient care. Multiple linear regression (MLR) was used to analyze the prediction of delirium care behaviour among nurses. The level of significance was set at 0.05, a two-tailed test.

## 3. Results

### 3.1. Demographic Characteristics 

The study sample consisted of 342 participants, a majority of whom were female nurses (86.5%). Over half of the participants (53.2%) were within the age range of 21–30 years. The respondents came from a range of specialist backgrounds, including surgical, medical, gynecological, geriatric, and orthopedic units, with the majority coming from surgical and medical units. Other units included emergency, palliative, rehabilitation, and combined units. The proportions were fairly similar to the overall proportions of nurses working in different units. The majority of the participants (78.4%) had obtained a bachelor’s degree, while 15.5% had attained a master’s degree or above. In terms of the number of years worked in the field, 45% of the respondents had between 1 and 5 years of clinical experience, while 17% had worked in the profession for over 16 years. In terms of experience in delirium care, the majority of nurses (87.4%) reported that they had cared for delirium patients. Among the respondents, 278 (80.1%) had not attended any formal delirium training courses, while 48 (14%) had participated in courses of less than 5 h and only 14 (4.1%) had attended courses of more than 10 h (Table 1).

### 3.2. Delirium Knowledge, Delirium Care Behaviours, and Factors Affecting Assessments of Delirium

Descriptive statistics were employed to examine the mean and standard deviation values of the variables. The scores obtained on the delirium knowledge questionnaire were notably high for the questions ‘Delirium is associated with confusion’ and ‘Delirium is associated with disturbed sleep cycles’. Conversely, the questions with the lowest scores were ‘Indwelling catheter can reduce the chance of delirium’ and ‘Constipation is not a cause of delirium’. With regard to behaviours, the highest scores were achieved for the statements ‘I will remove dangerous objects around delirious patients’ and ‘When the patient’s state of consciousness is abnormal’. The lowest scores for delirium care behaviours were registered for the items ‘I will discuss delirium prevention with the family’ and ‘I will use the delirium tool to assess delirium in my patients’. With regard to delirium care behaviours and factors affecting nurses’ delirium assessments, the raw scores for the three dimensions had to be standardized for comparison due to the uneven number of questions. Data were standardized to a total score of 100 for analysis. (Table 2). 

### 3.3. Correlations Between Delirium Knowledge, Delirium Care Behaviour, and Barriers to Delirium Assessments by Nurses

Pearson’s correlation was used to analyze the correlations between delirium knowledge, delirium care behaviour, and barriers to delirium assessments by nurses. The results showed a positive moderate correlation between delirium knowledge and delirium care behaviour scores, with R = 0.339 (*p* < 0.01). The correlation between delirium knowledge scores and barriers to implementation was 0.148 (*p* < 0.01), which was slightly positive and close to unrelated (Table 3).

Table 2 demonstrated that there was considerable variation in the means and standard deviations of the three dimensions of the barrier. To ensure that individual, organizational, and patient factors influencing the ability of nurses to perform delirium assessments were more effectively detected, repeated measures ANOVA was employed to compare the means of three sets of data for dependent samples of the same group [30,31].

In evaluating the appropriateness of conducting the Repeated Measures ANOVA, the assumption was determined using Mauchly’s spherical test, where the initial assumption was *p* > 0.05. The results demonstrated a value of W = 0.953 with a significance level of *p* = 0.000, indicating that the assumption of sphericity was not met. In order to account for the lack of sphericity, Greenhouse–Geisser and Huynh–Feldt corrections were employed [29,32]. The resulting F-value was 272.338, with a corresponding *p*-value of 0.000. This indicated that there were significant differences between the three factors, namely, personal, patient, and organizational factors (Table 4).

Given that the means of the three factors were statistically significant, a paired-*t* test was employed to determine whether there were notable discrepancies between the dimensions. To prevent the occurrence of a Type 1 error due to repeated testing, the corrected significance level was set at *p* < 0.05/3 = 0.016. This resulted in all three sets of paired data demonstrating statistically significant outcomes (*p* < 0.001) (Table 5).

A multiple regression analysis was conducted to predict and explain the impact of the independent variables on the dependent variables. In this analysis, the dependent variable was delirium care behaviour. In order to consider the independent variables, we referred to similar studies and also to those variables that exhibited significant correlations with the dependent variable in the present study, with a view to determining which variables should be included as independent variables. The following independent variables were identified: gender, age, education, years of clinical experience, the number of patients with delirium that were cared for, the number of hours spent on delirium care-related courses, knowledge of delirium, and the assessment of barriers to nursing care. To examine whether there was a covariance problem in the predicted variables in the regression analysis, the Variance Inflation Factor (VIF) and tolerance were examined. A VIF value of greater than 5 or a tolerance value of below 0.1 indicates the presence of multicollinearity among the independent variables. This implies that the explanatory variables are highly correlated [33]. Our analysis revealed that the VIF value ranged from 1.008 to 1.044, while the tolerance value ranged from 0.958 to 0.990. These findings suggest that multicollinearity was not a concern in this case.

Using the Stepwise Regression Method, the results indicated that years of clinical experience, the length of hours spent studying delirium care, a knowledge of delirium, and individual factors related to the delirium assessment could be included in the regression analysis with a significant difference of *p* < 0.001. By contrast, gender, age, education, patient factors, and total barrier scores could not be included in the regression analysis. The results of the regression analysis indicated that the four variables were able to fit the model and collectively explained 20.4% of the variance in delirium care behaviours, with an adjusted R² of 0.204 and an F-value of 22.527 (*p* < 0.001). In terms of the magnitude of explanatory power, delirium knowledge was found to have the greatest explanatory power for delirium care behaviours. The beta coefficient for delirium knowledge was 0.264 (95% CI: 0.474–1.019), which was higher than that for ‘years of working experience’, standing at 0.206 (95% CI: 1.125–3.158), and that for ‘duration of study hours’, standing at 0.103 (95% CI: 0.118–3.339). Ultimately, the beta coefficient for the personal factor was −0.239 (95% CI: −1.031–−0.432), indicating that the personal factor preventing nurses from conducting delirium assessments had a significant negative impact on behaviours related to delirium care (Table 6).

## 4. Discussion

### 4.1. Knowledge of Delirium Care and the Behavioural Status of Nurses

With regard to knowledge of delirium care, the mean value of this variable in the present study was 77.9 points after standardization. This is comparable to the finding of Wang [34], who used the same research instrument. Furthermore, the results were comparable to those of a study conducted in New Zealand, in which the nurses had a mean score of 80% for their knowledge [14]. This may be attributable to the fact that the study population comprised nurses from a range of ward settings, rather than solely from the critical care unit. A survey of 324 critical care nurses in Hong Kong, a city neighbouring Macau, revealed that the average scores for delirium knowledge ranged from 41.5% to 78.9% across different domains, with an overall mean of 65.9%. The highest score was observed for the predisposing and precipitating factors, while the lowest was seen for the assessment and detection factors [35]. This overall mean score is lower than the moderately high level of delirium knowledge among the Macau nurses. However, it is essential to note that the sample size, study design, and the hospital units differed between these studies, which could influence the comparability of the results.

The higher scores observed among general nurses in this study can be attributed to the pervasive implementation of the protocol “Enhanced Recovery after Surgery” across diverse medical specialties, including orthopedics, cardiothoracic surgery, obstetrics, and gynecology, as well as in general surgery units. The protocol consists of an important series of perioperative management methods that are optimally integrated to reduce surgical stress response and the occurrence of complications and promote functional recovery. The implementation of the protocol in all hospitals in Macau has led to increased awareness among nurses about delirium after surgery. However, training in delirium should extend beyond theoretical knowledge and protocols to encompass practical applications and the utilization of case vignettes to assess clinical reasoning [36]. Programmes that prioritize practice and bedside coaching, with an emphasis on doing, meaning, and knowing, have been demonstrated to be effective in sustaining and enhancing nurses’ knowledge and skills in their daily practice.

### 4.2. Caring Behaviours of Nurses for Delirium Patients

The provision of care for patients with delirium represents a pivotal aspect of nursing practice, and the care behaviours of nurses for delirium patients warrant further investigation. This is particularly the case in acute care settings, including medical, surgical, and acute geriatric units. The results indicated that the mean value of delirium care behaviour was 75.01%. A study conducted in a regional hospital in Taiwan revealed a markedly lower mean score of 63.5% (SD = 12.1) [34]. As indicated in the questionnaire, the responsibilities of nurses in delirium management include cognitive interventions, effective communication with family members, and the use of a delirium tool to assess patients. The ability to utilize a delirium assessment tool to evaluate patients is crucial. Siddiqi [37] posited that the use of delirium screening tools has the potential to reduce the incidence of delirium among hospitalized patients. Hospitals may consider acquiring and using delirium screening tools, such as the Confusion Assessment Method (CAM) or the Intensive Care Delirium Screening Checklist (ICDSC). The CAM is an effective tool for identifying and recognizing delirium in both clinical and research settings [38]. Moreover, it is challenging to assess patients with delirium, dementia, and depression as the three may occur simultaneously in the same patient. The Memorial Delirium Assessment Scale could be utilized as it is a valuable tool for diagnosing delirium and effectively differentiates delirium from dementia [39]. It is only through an accurate and prompt assessment that delirium can be correctly diagnosed, allowing for the subsequent implementation of appropriate care management.

### 4.3. Barriers to Assessing Delirium Patients

In our study, the patient-related factor emerged as the most significant barrier to the performance of delirium assessments by nurses, followed by organizational and person-related factors. The three most significant barriers at the patient level were identified as the difficulty of assessing patients with dementia, patients with severe neurological impairment and communication difficulties, and patients who were unwilling to cooperate with the assessor. It was observed that there can be a certain degree of overlap between dementia and delirium, with some patients exhibiting symptoms of both conditions. In order to overcome these barriers, healthcare professionals must dedicate more time to observing the characteristics of the patients’ seizures and the changes in the patients’ condition, with the aim of providing a comprehensive and holistic assessment [40]. Nurses can face significant difficulties in assessing delirium in patients with dementia, neurological impairment, and communication difficulties. Despite the availability of assessment tools, the difficulties in accurately diagnosing delirium may lead to delayed or inappropriate treatment, which can ultimately lead to adverse patient outcomes [37]. This can result in prolonged hospital stays, increased healthcare costs, and poor patient outcomes. To overcome these challenges, nurses must use a combination of assessment tools and strategies, including indirect measures based on their professional experience, to accurately diagnose delirium. They also need to be aware of the complexity of delirium and the potential for misdiagnosing or undertreating these patients [41]. It is essential to gain a comprehensive understanding of the subtle differences in delirium assessments in order to provide effective care for patients with complex conditions.

In terms of organizational barriers, our respondents indicated that some of the ward units lacked a process-specific assessment related to delirium, formal training related to delirium assessment tools, and the time required to conduct delirium assessments due to a busy clinical schedule. In addition to providing standardized education on the signs and symptoms of delirium, hospitals can also support nurses by implementing consistent delirium assessment tools and protocols. A systematic review and meta-analysis demonstrated that both the CAM and CAM-ICU are tools that can be readily administered, as they exhibit high sensitivity and specificity. Nevertheless, it is not possible to replace clinical judgement with these objective measures [42]. It is recommended that hospitals implement the CAM or other standardized tools to ensure that nurses employ a consistent and reliable method to assess delirium. The most commonly used delirium assessment tools will be made available in an electronic format, thus helping nurses to work more efficiently and enabling the straightforward retrieval of clinical data. Different ward units should establish clear policies and protocols for the management of delirium, including guidelines for the administration of medication, fluid therapy, and nonpharmacological interventions. Moreover, the implementation of delirium assessment process audit reports can be an effective approach and one that enables evidence to be collected on nurses’ knowledge, delirium awareness, assessment capabilities, and patient management [43]. The formation of a delirium care team or a dedicated delirium care department will facilitate the development of nurses’ judgement and their holistic management of delirium. Additionally, it is vital to provide opportunities for nurses to share their experiences and concerns, as well as to offer support and resources to nurses facing the emotional the challenges of caring for delirium patients.

In terms of personal barriers, the respondents indicated that a lack of knowledge regarding delirium identification and diagnosis, the failure to conduct timely and repeated assessments of delirium for patients, and uncertainty about the results of one’s own assessment were the primary barriers. In order to address these personal barriers, it would be beneficial to consider the implementation of simulation-based training in addition to traditional education and training programmes. Such training would be of assistance to nurses in that the assessment and management of delirium would be practised in a controlled environment, thereby enabling the nurses to develop their skills and confidence in a low-stakes setting. Moreover, mentorship and coaching can also be instrumental in addressing personal barriers to delirium care. Mentoring can facilitate the development of skills and confidence in nurses through the establishment of a supportive relationship [44]. Moreover, encouraging self-reflection and self-assessment can assist nurses in identifying areas for personal growth and development [45]. By reflecting on their own practice and assessing their skills and knowledge in delirium assessment and management, nurses will be able to identify areas in which they require further education and training. Furthermore, engaging in self-reflection and self-assessment can help nurses to develop a heightened sense of autonomy and self-efficacy, which can ultimately result in enhanced patient outcomes [46]. The implementation of these solutions will enable nurses to overcome personal barriers to delirium care and provide exemplary care to patients with delirium.

### 4.4. Analysis of Variables to Predict Delirium Care Among Nurses

Our results showed that the important factors influencing the delirium care behaviour of nurses were years of clinical experience, the length of study on courses related to delirium care, a knowledge of delirium care, and personal factors influencing nurses’ delirium assessments. The adjusted R^2^ was 0.204, *p* < 0.001, which meant that delirium care behaviour could only be partially predicted, and the four variables mentioned above still had some influence on delirium care behaviour.

The findings emphasized the pivotal role of multiple factors in shaping nurses’ delirium care practices. The significant positive correlation between years of clinical experience and delirium care behaviour indicates that nurses with greater experience may demonstrate enhanced confidence and competence in assessing and managing delirium, which may ultimately result in improved patient outcomes. A systematic review indicated that the work environment of nurses, including their experiences, is associated with patient outcomes that are sensitive to nursing care. Delirium care constitutes one of the five nurse-sensitive patient outcomes [47]. The duration of participation in formal courses related to delirium care and the level of knowledge acquired in such courses are associated with more effective delirium care practices among nurses. The aforementioned evidence suggests that formal training in delirium care is essential to ensure the delivery of high-quality care in relation to nurse caring behaviours. The findings align with that of [48], indicating that level of education is a significant predictor of nurses’ perceptions of their caregiving behaviours. The term ‘caring behaviour’ as used in this study encompasses patient advocacy, respectfulness, and the provision of optimal nursing care for patients suffering from delirium. It is recommended that educational programmes for post-registration nurses facilitate the critical examination of both unprofessional and caring behaviours amongst learners. The design of an educational programme for newly qualified nurses therefore encompasses a number of key elements, including a code of conduct, professional accountability, a reporting mechanism, and holistic patient care. However, the inverse relationship between personal factors and delirium care behaviour is a cause for concern. The failure to provide timely assessments of patients with delirium, a lack of confidence, and the belief that a delirium assessment will not improve the patient’s prognosis can have a significant impact on the ability of nurses to provide high-quality care to patients with delirium. In light of the above factors, it is recommended that the continued educational intervention should focus on assessing delirium and be based on empirical findings. The content of the education should be tailored to the specific ward setting, the patient population, and the specific delirium screening tool used [49]. Furthermore, a recent scoping review has highlighted that education interventions should focus on enhancing nurses’ perceptions of delirium, boosting their confidence and improving their knowledge of managing delirium patients [50].

This study is not without its limitations. A cross-sectional study was used to collect research data through convenience sampling, a method which affects the generalizability of the results obtained. This study was conducted using an electronic questionnaire; thus, the respondents could have searched for the correct answers before answering, which may have affected the results of the study. As the majority of the respondents had a bachelor’s degree and a master’s degree or higher, the findings of this study may not be applicable to other nurses with lower levels of education. 

## 5. Conclusions

The occurrence of delirium can give rise to a number of deleterious consequences. This study is the first of its kind to examine in detail the barriers that influence nurses’ behaviour from individual, patient, and organizational perspectives. It is of the utmost importance that nurses receive comprehensive training in the early assessment, risk identification, and appropriate care of patients with delirium. This will ensure the maintenance of patient safety and quality of care. With regard to knowledge of delirium care, the findings indicated that nurses in Macau demonstrated a moderate-to-high level of knowledge. With regard to the implementation of delirium care behaviours, the findings indicated relatively low scores in delirium assessment behaviours. A comparison of factors that prevented nurses from performing delirium assessments revealed significant differences. The personal factor was identified as the strongest predictor, followed by the organizational and patient factors. Finally, the results demonstrated that nurses’ clinical experience, knowledge of nurse delirium care, and length of study in delirium care-related courses, as well as barriers relating to personal factors, predict nurse delirium care behaviours.

## Figures and Tables

**Table 1:** Participants’ demographic characteristics.

Variable	Category	n (%)
Gender	Male	36 (10.5)
	Female	306 (89.5)
Age	21–30 years old	182 (53.2)
	31–40 years old	101 (29.5)
	41–50 years old	45 (13.2)
	51–60 years old	14 (4.1)
Education	College graduate	13 (3.8)
	Bachelor’s degree	268 (78.4)
	Postgraduate diploma	8 (2.3)
	Master’s degree and above	53 (15.5)
Work specialities	Medical	97 (28.4)
	Surgical	95 (27.38)
	Orthopaedic	25 (7.3)
	Gynaecological	19 (5.6)
	Gerontological	15 (4.4)
	Others	91 (26.6)
Work experience in years	1–5	154 (45)
	6–10	84 (24.6)
	11–15	46 (13.5)
	≥16	58 (17.0)
Delirium care experience	No	43 (12.6)
	Yes	299 (87.4)
Total number of delirium patients cared for in the past year	0	35 (10.2)
	1–5	169 (49.4)
	6–10	59 (17.3)
	>10	79 (23.1)
Total hours of formal training in delirium care	0	274 (80.1)
	<5	48(14.0)
	6–9	6 (1.8)
	>10	14 (4.1)

**Table 2:** Scores on delirium knowledge, delirium care behaviours, and factors affecting assessments of delirium.

Variables	Mean (SD)	Minimum	Maximum
Delirium Knowledge score	23.37 (+4.44)	0	30
Delirium Care Behaviours score	75.01 (+12.52)	20	100
(Standardized score of 100)			
-Prevention	76.96 (+12.78)	10	50
-Assessment	65.44 (+16.52)	5	25
-Care	80.68 (+14.24)	5	25
Total	75.01 (+12.52)	20	100
Factors affecting assessments of delirium			
(Standardized score of 100)			
-Personal factors	58.50 (+11.41)	7	35
-Patient factors	77.32 (+14.41)	5	25
-Organizational factors	73.37 (+13.83)	8	40
Total	69.15 (+9.75)	20	100

**Table 3:** Correlation matrix of delirium knowledge, delirium care behaviour, and barriers to assessments.

	Delirium Knowledge	Care Behaviour	Barriers
Delirium knowledge	1		
Care behaviour	0.339 **	1	
Barriers	0.148 **	−0.008	1

** *p* < 0.01.

**Table 4:** Interpretation of Mauchly’s Test of Sphericity.

Mauchly’s Test of Sphericity				
Intra-subject effects	Mauchly’s W	Approximate chi-square test	df	*p*
Factor 1	0.953	16.331	2	0.000
Intra-subject analysis of three dimensions of variability				
Source Factor 1		F		*p*
	Greenhouse–Geisser	272.338		0.000

**Table 5:** Pairwise comparisons of variables.

Pairing	Variable	Mean (SD)	Mean Difference	T-Value	*p*-Value
Pairing 1	Personal	58.50 (+11.42)	−18.82	−20.10	<0.001 **
	Patient	77.32 (+14.41)			
Pairing 2	Personal	58.50 (+11.42)	−14.87	−18.87	<0.001 **
	Organizational	73.37 (+13.83)			
Pairing 3	Patient	77.32 (+14.41)	3.95	4.82	<0.001 **
	Organizational	73.37 (+13.83)			

** *p* < 0.001.

**Table 6:** Results of the multiple linear regression analysis (n = 336).

Predictors	Standardized Coefficients	95% CI		T-Statistics	*p*-Value		
	Beta	Lower	Upper			Tolerance	VIF
Personal factor	−0.239	−1.031	−0.432	−4.806	0.000 **	0.958	1.044
Delirium knowledge	0.264	0.474	1.019	5.391	0.000 **	0.992	1.008
Years of experience	0.206	1.125	3.158	4.146	0.000 **	0.964	1.037
Hours of delirium courses	0.103	0.118	3.339	2.109	0.036 *	0.990	1.010
*R* = 0.463 *R*^2^ = 0.214 Adjusted *R*^2^ = 0.204 F = 22.527 *p* = 0.000							

* *p* < 0.05 ** *p* < 0.01.

## Data Availability

The data presented in this study are available on reasonable request from the corresponding author.
